# Quantifying Dissolved Transition Metals in Battery Electrolyte Solutions with NMR Paramagnetic Relaxation Enhancement

**DOI:** 10.1021/acs.jpcc.3c01396

**Published:** 2023-05-16

**Authors:** Jennifer P. Allen, Christopher A. O’Keefe, Clare P. Grey

**Affiliations:** † Yusuf Hamied Department of Chemistry, University of Cambridge, Lensfield Road, Cambridge CB2 1EW, U.K.; ‡ The Faraday Institution, Quad One, Harwell Science and Innovation Campus, Didcot OX11 0RA, U.K.

## Abstract

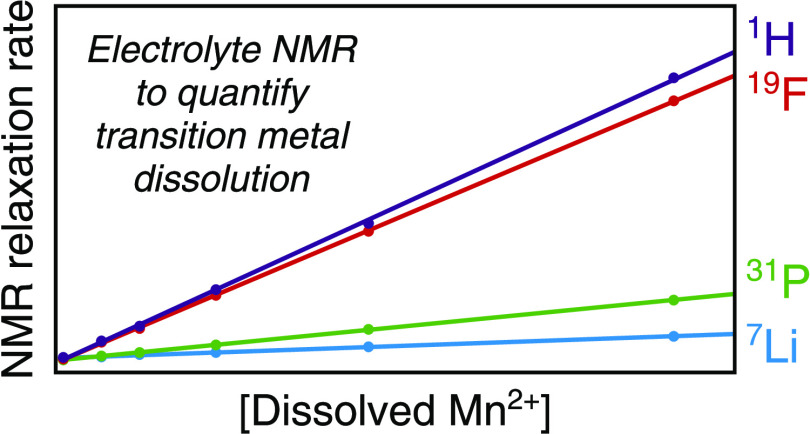

Transition metal dissolution is an important contributor to capacity fade in lithium-ion cells. NMR relaxation rates are proportional to the concentration of paramagnetic species, making them suitable to quantify dissolved transition metals in battery electrolytes. In this work, ^7^Li, ^31^P, ^19^F, and ^1^H longitudinal and transverse relaxation rates were measured to study LiPF_6_ electrolyte solutions containing Ni^2+^, Mn^2+^, Co^2+^, or Cu^2+^ salts and Mn dissolved from LiMn_2_O_4_. Sensitivities were found to vary by nuclide and by transition metal. ^19^F (PF_6_^–^) and ^1^H (solvent) measurements were more sensitive than ^7^Li and ^31^P measurements due to the higher likelihood that the observed species are in closer proximity to the metal center. Mn^2+^ induced the greatest relaxation enhancement, yielding a limit of detection of ∼0.005 mM for ^19^F and ^1^H measurements. Relaxometric analysis of a sample containing Mn dissolved from LiMn_2_O_4_ at ∼20 °C showed good sensitivity and accuracy (suggesting dissolution of Mn^2+^), but analysis of a sample stored at 60 °C showed that the relaxometric quantification is less accurate for heat-degraded LiPF_6_ electrolytes. This is attributed to degradation processes causing changes to the metal solvation shell (changing the fractions of PF_6_^–^, EC, and EMC coordinated to Mn^2+^), such that calibration measurements performed with pristine electrolyte solutions are not applicable to degraded solutions—a potential complication for efforts to quantify metal dissolution during *operando* NMR studies of batteries employing widely-used LiPF_6_ electrolytes. *Ex situ* nondestructive quantification of transition metals in lithium-ion battery electrolytes is shown to be possible by NMR relaxometry; further, the method’s sensitivity to the metal solvation shell also suggests potential use in assessing the coordination spheres of dissolved transition metals.

## Introduction

Understanding and preventing degradation in lithium-ion cells is vital to extending their lifetimes and increasing their applications. There are various causes of cell degradation, but one mechanism is the dissolution of transition metal ions from cathode materials (*e.g.*, Li[Ni*_x_*Mn*_y_*Co_1–*x*–*y*_]O_2_ or NMC) into the electrolyte solution, followed by the deposition of those metal ions at the anode.^1−6^ Although metal dissolution causes negative effects at the positive electrode due to the loss of active material and reconstruction of the cathode surface, the majority of transition metal-related capacity losses from NMC and similar cathode materials arise from metal deposition and reaction at the negative electrode.^7−13^ Once deposited at the anode, transition metals disrupt the solid electrolyte interphase (SEI), induce further electrolyte decomposition, consume cyclable lithium, and contribute to SEI thickening (with varying severity).^7−30^ The extent of dissolution from NMC, LiMn_2_O_4_, and LiNi_0.5_Mn_1.5_O_4_, *ex situ* (*e.g.*, from cathode powders stored in electrolyte solutions) or in cells, varies widely depending on factors, including storage time,^9,14,31−33^ cycle number,^33−39^ electrolyte composition,^7,34,40−49^ use of cathode coatings,^37,50−56^ upper cutoff potential,^8,12,13,29,36,38,40,44,57−61^ and temperature.^9,12,31,33,38,57^

Dissolved transition metals in electrolyte solutions can be quantified in several ways. The two most commonly used methods are inductively coupled plasma optical emission spectroscopy (ICP-OES)^14,31,33,38,40,62−64^ and ICP mass spectrometry (ICP-MS),^23,46−48,65−69^ while ion chromatography,^61,70−72^ total reflection X-ray fluorescence,^73−75^ and ultraviolet–visible spectroscopy^62,63,76−78^ have also been applied. Electrochemical methods may also be used; differential pulse polarography experiments^34,79^ and electrodeposition onto a rotating ring disk electrode^65^ have been used to quantify Mn^2+^ dissolved from LiMn_2_O_4_.

The above methods provide precise quantification of the metal concentration in solution; however, most of these destroy or alter the sample, either because the measurement itself is destructive or because chemical agents are added during sample preparation. By contrast, nuclear magnetic resonance (NMR) spectroscopy is a nondestructive technique that has been used to study lithium-ion cells because of its chemical specificity and ability to provide information about both structure and dynamics. NMR-active nuclei present in most lithium-ion cells include ^1^H, ^7^Li, ^13^C, ^17^O, ^19^F, and ^31^P, among others. Solid-state NMR has been used to study the electrode materials^80−86^ and electrode–electrolyte interfaces,^84,87−94^ while solution NMR has been used to study the electrolyte decomposition products,^95−101^ transport properties,^102−110^ and solvation structure.^109,111−116^*In situ* and *operando* NMR experiments have even been developed to probe chemical and electrochemical processes occurring in custom cells.^82−86,105,110,117−119^ Although deuterated solvents may be added to electrolytes for solution NMR (irreversibly altering the sample and complicating potential *operando* analysis if deuterated electrolytes are not available), this is not strictly necessary as these solvents may be incorporated instead by using a solvent capillary or coaxial tube.

Because dissolved transition metals are paramagnetic, with very rapid nuclear relaxation rates,^80,120^ NMR is not well suited for the direct (*e.g.*, ^61^Ni, ^55^Mn, ^59^Co) measurement of transition metal dissolution and deposition. However, the magnetic properties of other nuclei in the same sample are affected by the presence of paramagnetic species, and NMR experiments focusing on these other nuclides can reveal information about the metals, including their oxidation state and coordination environment. A key effect of paramagnetic species on NMR measurement is relaxation enhancement, where the unpaired electrons of the paramagnetic species create fluctuating magnetic fields that drive efficient relaxation of nearby nuclei.^121−124^ Relaxation times in both the longitudinal dimension (*T*_1_, along the applied magnetic field *B*_0_) and transverse dimension (*T*_2_, perpendicular to *B*_0_) are affected by this interaction. Critically, the relaxation enhancement is proportional to the concentration of paramagnetic species,^121^ so measurement of the NMR relaxation rate should enable quantification of the dissolved metals. NMR field-cycling relaxometry has recently been used to quantify dissolved Mn^2+^ in wine, an example of an application of the method to an “electrolyte” containing multiple dissolved components.^125^ The properties and equations governing paramagnetic relaxation enhancement are described in detail in the Discussion section.

If nondestructive quantification is possible with NMR relaxometry, this would not only preserve samples for later analysis but also facilitate *operando* metal quantification with NMR. *Operando* quantification of metal dissolution in a lithium-ion cell has been achieved with ICP-MS coupled to an electroanalytical flow cell,^68^ but this method is invasive, requiring the electrolyte to be removed from the cell; thus, the effects of electrolyte degradation and transition metal deposition on cell performance are not captured. The use of relaxation-weighted magnetic resonance imaging (MRI) could be used not only to noninvasively quantify metal dissolution but also to visualize the distribution of dissolved transition metals in a cell, by mapping relaxation rates across the electrolyte volume (where faster relaxation rates indicate larger metal concentrations). Such work has already been successfully performed to examine Cu^2+^ electrodissolution into an aqueous Na_2_SO_4_ electrolyte solution.^126^

This work analyses the relaxation rates of electrolyte solutions comprising LiPF_6_, carbonate solvents, and μM–mM quantities of Ni^2+^, Mn^2+^, Co^2+^ (which may dissolve from NMC cathodes), or Cu^2+^ (which may dissolve from copper current collectors), in order to establish whether relaxometry provides a viable method to quantify metal dissolution. This work follows from our previous studies: in the first, we explored the use of bulk magnetic susceptibility shifts to identify the oxidation states and concentrations of dissolved paramagnetic ions.^127^ In the second, we examined the line broadening induced by paramagnetic ions in pristine and degraded battery electrolytes,^128^ describing methods to mitigate this. Here, we analyze relaxation phenomena in greater detail, measuring and analyzing both transverse and longitudinal relaxation rates; we show that they are highly sensitive to the presence of Mn^2+^, especially with ^19^F and ^1^H measurements. Storage of LiMn_2_O_4_ powder with the electrolyte solution at ∼20 °C shows that Mn(TFSI)_2_ is indeed representative of Mn^2+^ dissolved from cathode materials, and metal quantification was successful. However, the quantification was far less accurate in a similar sample that had been stored at 60 °C, indicating that the species produced during the thermal degradation processes may change the transition metal coordination sphere and thereby reduce the applicability of the NMR calibration data acquired with the pristine electrolyte solutions. While potential *operando* applications face some complications (outlined in this work), *ex situ* relaxometry may be a valuable tool to nondestructively quantify dissolved metals in battery electrolyte solutions. Notably, the dependence of relaxation rates on metal solvation suggests that NMR relaxometry may also be applied to probe the coordination of dissolved metals to electrolyte species.

## Methods

### Preparation of Electrolyte Solutions

Electrolyte solutions comprised 1 M LiPF_6_ in 3:7 ethylene carbonate (EC)/ethyl methyl carbonate (EMC) (v/v), a standard composition that was sourced premixed (soulbrain MI PuriEL R&D 280). Trifluoromethanesulfonimide (TFSI) salts were used to simulate dissolved transition metals and were dried under vacuum at 100 °C before use: Mn(TFSI)_2_ (Tokyo Chemical Industry UK Ltd., >97.0% and Solvionic, 99.5%), Co(TFSI)_2_ (Alfa Aesar, ≥95.0%), Ni(TFSI)_2_ (Alfa Aesar, ≥97%), and Cu(TFSI)_2_ (purchased as the hydrate, Sigma-Aldrich).

### NMR Measurements of Electrolyte Solutions

NMR relaxation measurements were performed at ∼25 °C on a Bruker Avance III HD 300 MHz spectrometer using a Bruker double-channel MicWB40 probe. *T*_1_ measurements were acquired using the inversion recovery pulse sequence; *T*_2_ measurements were acquired using the Carr–Purcell–Meiboom–Gill (CPMG) pulse sequence^129,130^ with echo spacings of τ = 2 ms (with the exception of the ^19^F *T*_2_ measurement of 8.0 mM Mn^2+^, where τ = 1 ms was used, due to very rapid relaxation). NMR spectra were acquired using a Bruker Avance III HD 500 MHz spectrometer equipped with a broadband observe (BBO) probe; NMR tubes contained sealed C_6_D_6_ capillaries for field locking. In all cases, NMR tubes were filled in an argon glovebox and sealed with J-Young valves or with poly(tetrafluoroethylene) tape over the cap. Measurements were conducted shortly after solution preparation, and no significant changes in relaxation rates were detected after some solutions were stored outside the glovebox for ∼4 days.

### LiMn_2_O_4_ Storage Experiment

To study Mn dissolution from electrode materials, 3 g LiMn_2_O_4_ was combined with 7 mL of the electrolyte solution and stored in an aluminum bottle under argon for 88 days at ∼20 °C or 77 days at 60 °C. The solution was then centrifuged and the supernatant was analyzed with NMR and ICP-OES. ICP-OES samples were prepared *via* the addition of trace metal grade nitric acid, and measurements were carried out using an iCAP 7400 Duo ICP-OES Analyzer (Thermo Fisher Scientific).

## Results

**Figure 1:**
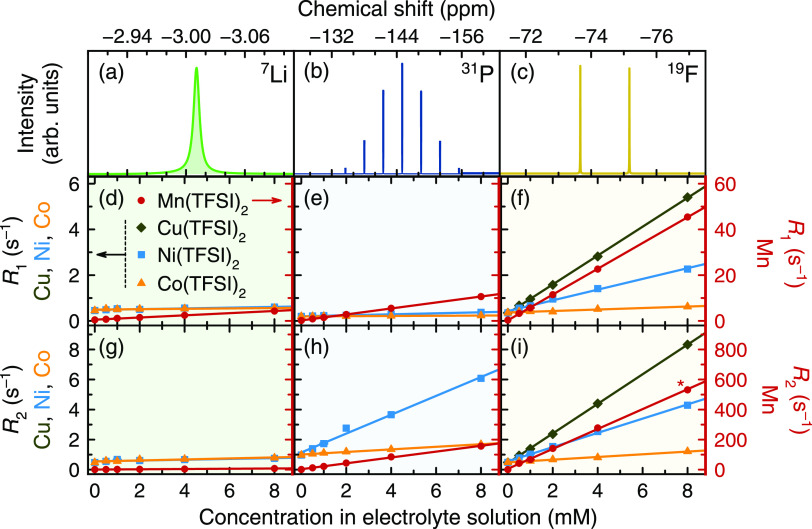
(a–c) ^7^Li, ^31^P, and ^19^F representative NMR spectra of diamagnetic 1 M LiPF_6_ in 3:7 EC/EMC (v/v) and (d–f) longitudinal and (g–i) transverse relaxation rates of electrolyte solutions containing dissolved Mn(TFSI)_2_, Cu(TFSI)_2_ (^19^F only), Ni(TFSI)_2_, or Co(TFSI)_2_. Only the spectral region showing the PF_6_^–^ is shown in the ^31^P and^19^F spectra; the observed multiplets arise from the ^31^P–^19^F *J*-coupling. Relaxation rates of solutions containing Cu(TFSI)_2_, Ni(TFSI)_2_, and Co(TFSI)_2_ are plotted on the left *y* axes, while relaxation rates of solutions containing Mn(TFSI)_2_ are plotted on the right *y* axes, as indicated by the arrows in panel (d). Error bars at 1 mM indicate the standard deviation of three measurements on the sample; they are difficult to visualize at this scale, but all 20 are <3%. The asterisk at 8 mM Mn^2+^ in panel (i) indicates the use of a reduced echo spacing of τ = 1 ms due to very fast relaxation. An expanded view of the ^7^Li and ^31^P relaxation data in panels (d), (e), (g), and (h) is provided in the Supporting Information (Figure S1).

Figure 1 shows diamagnetic ^7^Li, ^31^P, and ^19^F NMR spectra of LiPF_6_/EC/EMC solutions and relaxation measurements of solutions containing 0–8 mM dissolved Mn^2+^, Ni^2+^, and Co^2+^. ^19^F measurements are also shown for solutions containing Cu^2+^. Relaxation time constants are presented as relaxation rates in s^–1^, referred to as *R*_1_ (1/*T*_1_) and *R*_2_ (1/*T*_2_). An expanded view of the ^7^Li and ^31^P relaxation is provided in the Supporting Information (Figure S1). In all cases, the relaxation rates increase with the transition metal concentration. Generally, the relaxation rates follow the trend ^19^F > ^31^P > ^7^Li. Notably, relaxation rates in Mn^2+^-containing samples are 1–2 orders of magnitude faster than those in Cu^2+^-, Ni^2+^-, or Co^2+^-containing samples. Linear regression analysis shows that better fits are yielded for data with larger relaxation changes: the only datasets with *R*^2^ < 0.99 are those where very little change in relaxation is observed, namely, results from ^7^Li NMR and results from Co^2+^-containing samples. The relationship between gradient and *R*^2^ for each nucleus is shown in Figure S2.

**Figure 2:**
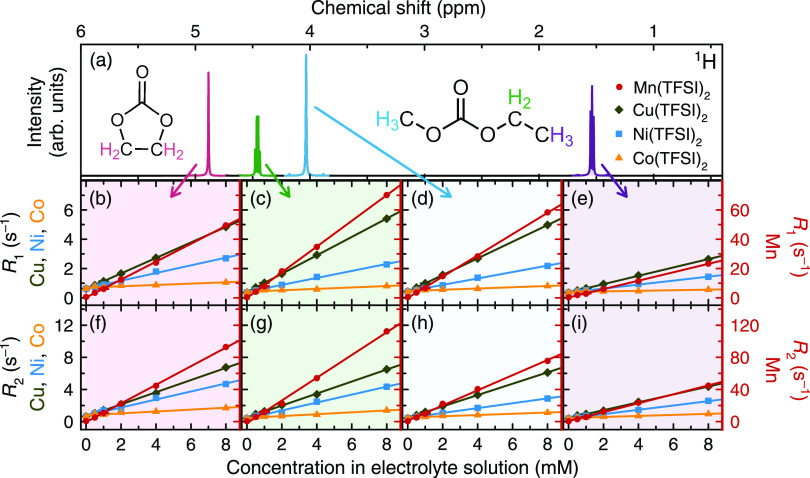
(a) Representative ^1^H NMR spectrum of diamagnetic 1 M LiPF_6_ in 3:7 EC/EMC (v/v) and (b–e) longitudinal and (f–i) transverse relaxation rates of electrolyte solutions containing dissolved Mn(TFSI)_2_, Cu(TFSI)_2_, Ni(TFSI)_2_, or Co(TFSI)_2_. Relaxation rates of solutions containing Mn(TFSI)_2_ are plotted on the right *y* axes. The multiple relaxation rates arise from the different ^1^H environments in the solvent system, indicated in panel (a). Error bars at 1 mM indicate the standard deviation of three measurements on the sample; while difficult to visualize at this scale, all 32 are <6%.

Figure 2 shows ^1^H relaxation rates of electrolyte solutions containing added transition metal ions. The four longitudinal and transverse relaxation rates correspond to the four ^1^H environments in the solvent system, indicated in Figure 2a. The ^1^H relaxation measurements show linearly increasing relaxation rates, with Mn^2+^ inducing the greatest change. Among the three EMC environments, the slowest-relaxing ^1^H environment is the CH_3_ of the ethyl group (Figure 2e,i), which shows considerably slower relaxation rates than the ethyl CH_2_ (Figure 2c,g) and the methyl group (Figure 2d,h). Linear regression analysis of the ^1^H data shows that it is mainly Co^2+^-containing samples that yield poorer fits, while among the 24 Ni^2+^, Cu^2+^, and Mn^2+^ datasets, the smallest *R*^2^ value is 0.986. The relationship between gradient and *R*^2^ is shown in Figure S2, which also contains data for ^7^Li, ^19^F, and ^31^P relaxation.

**Figure 3:**
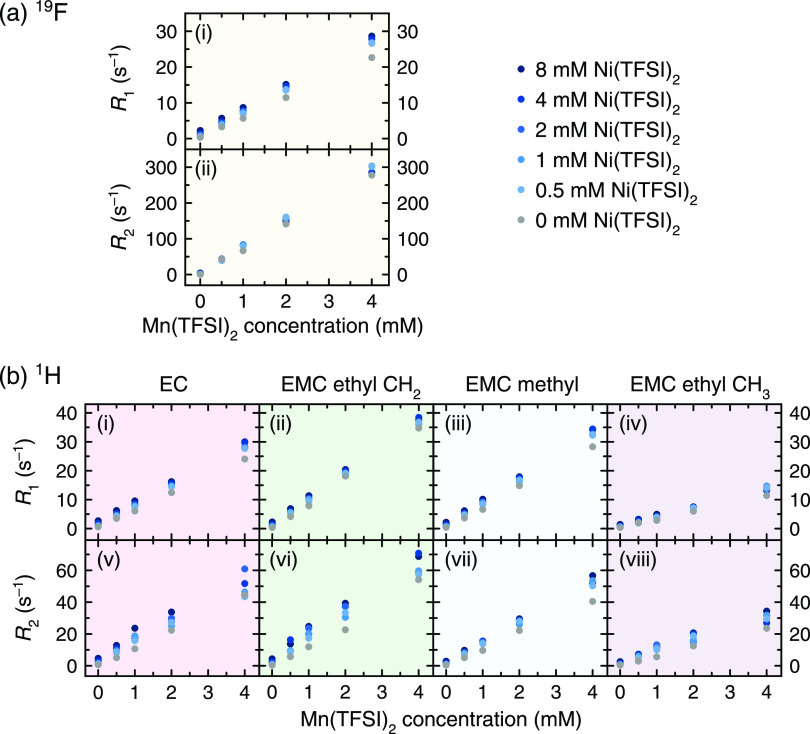
(a) ^19^F and (b) ^1^H longitudinal and transverse relaxation rates of 1 M LiPF_6_ in 3:7 EC/EMC (v/v) electrolyte solutions containing both dissolved Mn(TFSI)_2_ (concentrations shown on the *x* axis) and Ni(TFSI)_2_ (concentrations indicated by the color of the points). The multiple relaxation rates in the ^1^H NMR arise from the different ^1^H environments in the solvent system.

Figure 3 shows ^19^F and ^1^H relaxation measurements of electrolyte solutions containing both Mn^2+^ (0–4 mM) and Ni^2+^ (0–8 mM) ions. This experiment was performed to assess the suitability of the relaxometry method to determine whether concentrations of multiple metals can be detected in solution. While some differences are visible between samples containing different Ni^2+^ concentrations, such differences are small, and the magnitude of relaxation rates are not always in the anticipated order of increasing Ni^2+^ concentration (see, for example, the EC transverse relaxation at the highest Mn^2+^ concentration).

**Figure 4:**
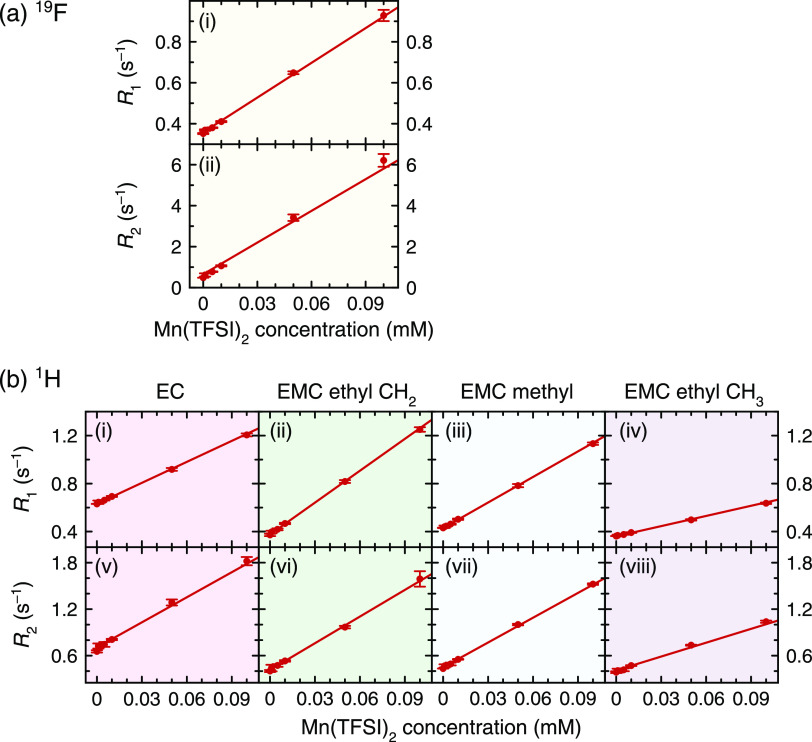
(a) ^19^F and (b) ^1^H longitudinal and transverse relaxation rates of 1 M LiPF_6_ in 3:7 EC/EMC (v/v) electrolyte solutions containing small amounts of dissolved Mn(TFSI)_2_ to determine the limit of detection of the method. The multiple relaxation rates in the ^1^H NMR arise from the different ^1^H environments in the solvent system.

Figure 4 shows ^19^F and ^1^H relaxation measurements of electrolyte solutions that contain very low concentrations of Mn^2+^ to estimate the method’s limit of detection (LoD). The measurements were performed at 0, 0.001, 0.005, 0.010, 0.050, 0.100, and 0.500 mM. (Although only 0–0.1 mM is shown in Figure 4, the lines of best fit include the 0.5 mM point, and the full 0–0.5 mM scale is shown in Figure S3.) These measurements show that the relaxation enhancement remains linear even at very low metal concentrations and that the method is highly sensitive to Mn^2+^. The ^19^F and ^1^H relaxation rates at 0, 0.001, and 0.005 mM Mn^2+^ are listed in Table S1. With both the 5σ criterion and the 5% error criterion, the LoD of the NMR relaxometry method is on the order of 0.001–0.005 mM (the limit for each of the 10 relaxation rates differs slightly).

**Figure 5:**
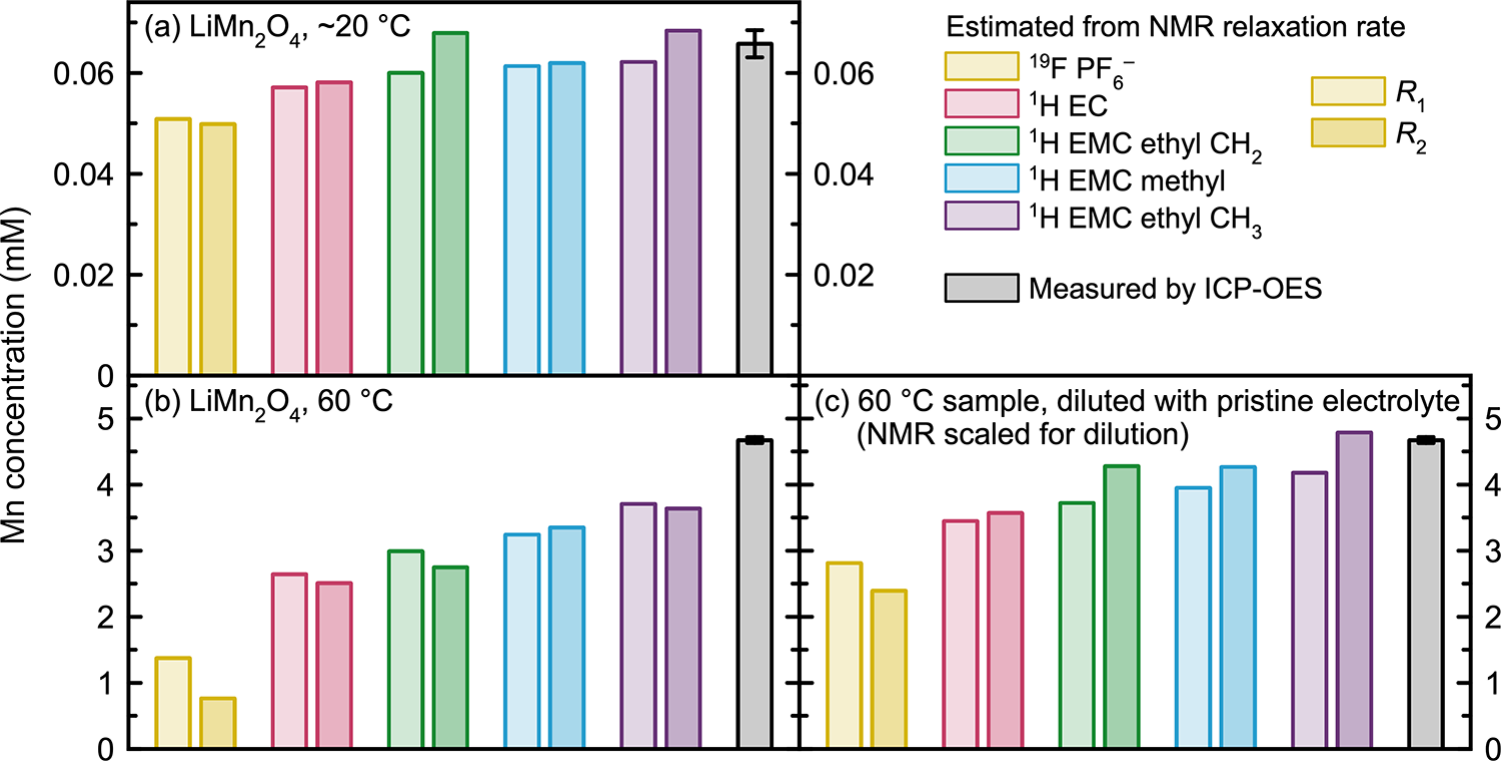
Estimated Mn concentrations after (a) ∼20 °C and (b, c) 60 °C storage of LiMn_2_O_4_ with 1 M LiPF_6_ in 3:7 EC/EMC (v/v), calculated from ^19^F and ^1^H longitudinal and transverse relaxation rates. In panel (c), the 60 °C sample was diluted 5× with the pristine electrolyte solution, and the resulting NMR concentrations were scaled by 5× for direct comparison with the results in panel (b). Black bars indicate the concentration measured by ICP-OES, with error bars indicating the standard deviation of three samples.

To determine whether these Mn(TFSI)_2_ results can be applied to Mn dissolved from battery materials, LiMn_2_O_4_ powder was soaked in the electrolyte solution and stored under argon at either ∼20 °C for 88 days or at 60 °C for 77 days. Figure 5 compares the estimates for quantities of dissolved metals obtained *via*^19^F relaxation, ^1^H relaxation, and by ICP-OES. Metals were quantified by relaxometry by using the linear fits from Figure 4. The sample stored at 60 °C was also diluted 5× with pristine electrolyte to determine whether more accurate estimates of Mn^2+^ concentration could be obtained from more dilute solutions, and the NMR results were scaled by 5× for direct comparison with the results obtained from the undiluted sample. Six months later, two additional samples were prepared; these samples were analyzed 9 months after the calibration dataset was measured, and the results are discussed in the Supporting Information (Figure S4).

For the sample stored at ∼20 °C for 88 days, the NMR estimates of Mn concentration are a good match with the ICP-OES measurement. However, for the 60 °C sample, all NMR estimates are considerably smaller than the ICP-OES measurement. The 60 °C sample contains almost 2 orders of magnitude more Mn than the 20 °C sample (4.67 ± 0.05 *vs* 0.066 ± 0.003 mM, respectively, from ICP-OES), and after dilution of the 60 °C sample with pristine electrolyte, the NMR estimates are more consistent with the ICP-OES result, although the concentration is still underestimated by NMR. Among the different NMR estimates for Mn concentration, the least accurate values (smallest estimated concentrations) are obtained from the ^19^F PF_6_^–^ relaxation rates, and the most accurate values are obtained when the concentration is estimated from the ^1^H EMC ethyl CH_3_ relaxation rates. The source of these deviations is addressed in the Discussion section.

## Discussion

In this work, the Solomon–Bloembergen–Morgan model^120−123,131^ of relaxation is adopted to provide a framework within which to discuss our results. Equations 1 and 2 describe the relaxation rates of nuclei bound to paramagnetic ions (*R*_1M_ and *R*_2M_ or 1/*T*_1M_ and 1/*T*_2M_, respectively), where the first terms describe through-space dipolar coupling of the nuclear and electron spins^120−122^ and the second terms describe the isotropic contact interaction.^120,121,123^ The paramagnetic relaxation enhancement is strongly affected by the ion–nucleus distance (1/*r*^6^) and the electron spin (*S*(*S* + 1)). Other values in eqs 1 and 2 include the permeability of vacuum (μ_0_), nuclear gyromagnetic ratio (γ_I_), electron spin *g*-factor (*g*_e_), Bohr magneton (μ_B_), Larmor frequencies for the nuclear spin (ω_I_) and electron spin (ω_S_), the hyperfine interaction constant (*A*/*ℏ*), and correlation times for the dipolar (τ_c_^dip^) and contact (τ_c_^con^) terms. The timescales of the fluctuating magnetic fields are quantified by the correlation times, which differ depending on the mechanism of coupling between the fluctuating field and the nuclear spin. For the dipolar term, τ_c_^dip^ is determined by the molecular rotation (τ_r_), electronic relaxation (τ_e_), and chemical exchange (τ_M_) correlation times, as shown in eq 3, while for the isotropic contact term, τ_c_^con^ is determined by τ_e_ and τ_M_, as shown in eq 4.^121^
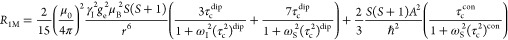
1
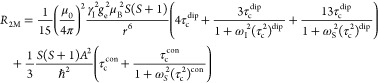
2
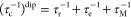
3
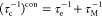
4The above equations describe the relaxation of the paramagnetic complex; however, in dilute solutions, most observed NMR nuclei are not in ions or molecules bound to paramagnetic ions. We must therefore take into account the probability of a nuclide being adjacent to a paramagnetic ion and thus the probability that it will undergo the relaxation processes described in eqs 1 and 2. Equations 5 and 6 show the paramagnetic relaxation enhancement *R*_1p_ and *R*_2p_, *i.e.*, the difference between the observed relaxation rates of a paramagnetic sample and its diamagnetic analogue (or *R*_1_ – *R*_1d_ and *R*_2_ – *R*_2d_).^121,132^ The relaxation enhancement is proportional to *f*_M_, the molar fraction of nuclei that are bound to paramagnetic metal ions. (There is also a dependence on chemical exchange, with timescale τ_M_, between the free species and the complex where the species is bound to the paramagnetic center; additionally, the transverse relaxation has a term that depends on the hyperfine shift Δω_M_.) The measured relaxation rates can therefore be related to eqs 1 and 2, which are derived for a defined complex with a finite lifetime (hence, paramagnetic relaxation effects due to free diffusion are ignored).
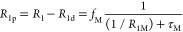
5
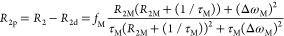
6Equations 1–6 show that the effect of dissolved transition metals on the relaxation rate is highly specific to the system, with a dependence on molecular motion (rotation and exchange), metal–nucleus distance, and the degree of transition metal coordination, among other factors.^121^ If the metal concentration is already known, the relaxometry result does not necessarily provide a clear indication of which oxidation state is dissolved; instead, a reliable calibration is required to interpret the relaxometry results. While differences in relaxometry results could indicate the dissolution of one oxidation state *vs* another, it could also indicate changes to the metal solvation shell. Although the interpretation of relaxometry results is by no means straightforward, given the multiple correlation times and interactions, we attempt to draw simple trends from the data, which will inevitably require a number of assumptions that we, when possible, explore.

Calibration curves of the NMR response to the presence of various transition metals are shown in Figures 1 and 2. In these figures, the limit of detection for the relaxometry method is observed to vary for each metal, nucleus, and environment. The method is most sensitive to paramagnetic species with more unpaired electrons (larger *S*) and slower electronic relaxation rates (longer τ_e_). Typical Mn^2+^ electronic relaxation rates are one or more orders of magnitude slower than those of Cu^2+^, Ni^2+^, or Co^2+^.^121^ The slower electron spin relaxation for Mn^2+^ arises from the lack of orbital angular momentum of the state with symmetry A, and this long τ_e_ results in Mn^2+^ inducing especially rapid nuclear relaxation.^120^ The relaxation enhancement from Mn^2+^ (3d^5^) is also expected to be stronger than that of Cu^2+^ (3d^9^), Ni^2+^ (3d^8^), and Co^2+^ (3d^7^) due to the greater number of unpaired electrons in Mn^2+^, which yields a significantly larger *S*(*S* + 1) term.

Nuclei that show a larger response to the metal concentration are likely in environments that are, on average, closer to the paramagnetic centers. The NMR nucleus may be closer to the metal binding site, as the dipolar relaxation enhancement of dissolved metals occurs through space with a 1/*r*^6^ dependence (eqs 1 and 2). Alternately, the fraction of the species coordinated to the transition metal may be larger (eqs 5 and 6). The ^7^Li, ^31^P, and ^19^F NMR results in Figure 1 indicate, reasonably, that the transition metal cations are found much closer to PF_6_^–^ than to Li^+^, presumably due to electrostatic interactions. The larger relaxation effect on ^19^F vs ^31^P nuclei is explained by the larger γ_I_ of ^19^F and also by the PF_6_^–^ structure, as P is surrounded by, and less accessible than, electron-rich F atoms. While paramagnetic relaxation enhancement applies to all nuclides in Figure 1, ^19^F NMR is therefore the most suitable of these for the measurement of dissolved transition metals, particularly Mn^2+^. As a result, the relaxation method may be readily applied to other electrolyte solutions containing fluorinated salts or solvents because they are observable with ^19^F NMR and should, in many cases, experience some electrostatic attraction to metal cations. ^7^Li NMR is least suitable for the measurement of dissolved transition metals due to cation repulsion. Although ^7^Li relaxation of Mn^2+^-containing samples shows strong linearity with metal concentration, we note that quadrupolar relaxation mechanisms may become increasingly more important at low metal concentrations, particularly for samples containing Ni^2+^ and Co^2+^, potentially resulting in nonlinear behaviour.

The ^1^H relaxation measurements (Figure 2) show the same general behavior as the ^7^Li, ^31^P, and ^19^F relaxation measurements (Figure 1). As EMC has three ^1^H environments, the differences among the relaxation rates reflect the relative distances between each environment and the coordinated metal ion (from the 1/*r*^6^ dependence of dipolar relaxation in eqs 1 and 2, although it is noted that binding to the metal ions is likely highly dynamic). Transition metal coordination to EMC occurs at the carbonyl oxygen: this is consistent with the observation of the slowest relaxation arising from the ethyl CH_3_ (Figure 2e,i) and the observation that the ethyl CH_2_ (Figure 2c,g) relaxes slightly faster than the methyl group (Figure 2d,h), since the ethyl CH_2_ hydrogens are, on average, closer to the C=O. (In the diamagnetic electrolyte solution, the three EMC ^1^H environments do not show equal relaxation rates; however, the trends stated here also apply to the relaxation enhancement from the diamagnetic baseline—the gradients of the lines of best fit in Figure 2 follow the order EMC ethyl CH_2_ > EMC methyl > EMC ethyl CH_3_.) These results echo the finding from a computational study, which suggested that Mn^2+^ would coordinate at the EMC carbonyl oxygen.^133^ These results are also consistent with theoretical and experimental studies of Li^+^ solvation, which show that metal coordination occurs preferentially at C=O for EC and linear carbonates.^112,134−139^

For all transition metal solutions and all nuclides, *R*_2_ is larger than *R*_1_. But whereas *R*_2_ is ∼1–2 times larger than *R*_1_ for ^1^H relaxation in all solutions and for ^19^F relaxation in Co^2+^-, Ni^2+^-, and Cu^2+^ containing solutions, *R*_2_ is an order of magnitude larger than *R*_1_ for ^19^F relaxation in Mn^2+^-containing solutions (Figures 1 and 2). This is likely due to the electronic relaxation time of Mn^2+^, which is slower than the electronic relaxation times of the other three metals.^121^ Dipolar relaxation processes for Mn^2+^ are therefore far more likely to be limited by a different correlation time. This would be relevant if, for example, there were multiple relaxation processes occurring; these could affect the *R*_1_ and *R*_2_ terms differently due to a motional component, which would only become apparent when the electronic relaxation time is sufficiently slow. Another potential reason that *R*_2_ may be much larger than *R*_1_ is the involvement of a contact term. The contact term in *R*_1M_ depends on the spectral density at the Larmor frequency of the unpaired electron spin, ω_S_, and the correlation time for the contact interaction, τ_c_^con^ (eq 1). Thus, in Mn^2+^-containing solutions, the *R*_1M_ contact term may approach 0 due to the large Larmor frequency of the unpaired electron spin and the slow electronic relaxation of the Mn^2+^ ion. By contrast, the *R*_2M_ contact term contains a frequency-independent component. If the *R*_2M_ contact term is large, and the *R*_1M_ contact term is negligible, then *R*_2_ can become much larger than *R*_1_.^123^ We also note that the exchange times may not be equal for all metals, as the interaction strengths with electrolyte components may differ, and this may also affect the observed relationship between *R*_2_ and *R*_1_. Unraveling the different mechanistic contributions to exchange processes may require measurements at different temperatures and magnetic field strengths, and such efforts are beyond the scope of this work.

In Figure 3, although the *R*_1_ measurements do show some differences between the samples containing different amounts of Ni^2+^, the difference between a sample containing 2 and 8 mM Ni^2+^ is too small to be quantified *viaR*_1_, and even falls within the error of *R*_2_ measurements. The relaxation rates should be additive at these low metal concentrations; for a solution containing both Ni^2+^ and Mn^2+^, *R*_1_ = *R*_1d_ + *R*_1p,Ni_ + *R*_1p,Mn_. That is, the total relaxation is expected to comprise a diamagnetic relaxation component (small), a component for the paramagnetic relaxation induced by Ni^2+^ (small), and a component for the paramagnetic relaxation induced by Mn^2+^ (large). For the samples used in this work, relaxation rates alone are not sufficient to distinguish the concentrations of Ni^2+^ and Mn^2+^ when both are in solution, due to the much larger effect of Mn^2+^. However, the low-concentration Mn^2+^ relaxation measurements in Figure 4 show the potential sensitivity of this method toward measuring realistic concentrations of dissolved Mn^2+^. Indeed, this is shown in Figure 5a, where the quantification of Mn dissolved from LiMn_2_O_4_ in the pristine electrolyte is a good match to the 0.066 ± 0.003 mM detected by ICP-OES. This suggests that the Mn dissolved from LiMn_2_O_4_ at ∼20 °C exists as Mn^2+^. Mn^3+^ has a different *S* and τ_e_ than Mn^2+^,^121^ in addition to likely having a different binding affinity for the electrolyte components; therefore, different *R*_1_ and *R*_2_ values would result if a significant fraction of dissolved Mn were present as Mn^3+^, likely requiring further study to explore Mn^3+^ relaxation phenomena. It is also promising that the calibration based on Mn(TFSI)_2_ could be successfully applied to a sample containing Mn dissolved from LiMn_2_O_4_, suggesting that the TFSI^–^ anion did not affect the calibration. While M(TFSI)_2_ salts were used in this work due to their high solubility and ready availability, we anticipate that any model compound in which the paramagnetic metal does not form a long-lived ion pair with the counterion could be used to quantify dissolution in samples retaining the same paramagnetic metal solvation shell as the calibration set.

After storage at 60 °C, the relaxation measurements provided a poor estimate of the actual Mn concentration (Figure 5b). This is not due to any nonlinear relaxation behavior at large Mn concentrations, as the 4.67 ± 0.05 mM concentration is well within the linear behavior shown in Figures 1 and 2 for 0–8 mM Mn^2+^. Although higher temperatures drive changes in relaxation behavior due to shortening of rotational and chemical exchange times and changes in electronic relaxation times, the 60 °C sample was cooled to ambient temperature before the relaxation measurements were performed; temperature is therefore not the cause of this discrepancy. While we should consider whether the poor match between relaxometry and ICP-OES results for the 60 °C sample occurred due to Mn^3+^ dissolution, the results from the ∼20 °C dissolution experiment show that Mn^2+^ is the dissolution species. Furthermore, our previous analysis of the high-temperature sample using NMR bulk magnetic susceptibility shifts to determine the metal oxidation state confirmed the presence of Mn^2+^ and not Mn^3+^ in the sample.^127^

The issue with relaxometry of the sample stored at 60 °C may therefore not be related to the Mn concentration or oxidation state but rather to the presence of degradation species in the electrolyte solution. This hypothesis is now explored. First, as shown in Figure 5c, the Mn concentration estimate was greatly improved by diluting the sample 5× with pristine diamagnetic electrolyte, *i.e.*, the estimate was more accurate when concentrations of degradation species were reduced by 5×. The paramagnetic relaxation enhancement *R*_1p_ and *R*_2p_ are directly proportional to *f*_M_, the molar fraction of nuclei bound to paramagnetic species (eqs 5 and 6). The fraction of nuclei that are bound to paramagnetic metal ions is a function of the number of metal ions, the metal solvation number, and the total number of nuclei in the sample. Equations 5 and 6 can be rewritten as eqs 7 and 8, in the form *y* = *mx* + *c*.
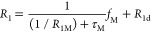
7
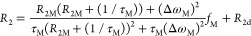
8In the pristine electrolyte calibration solutions (Figures 1 and 2), *f*_M_ was increased *via* the metal concentration, while the solution composition remained the same. At these relatively low concentrations, the strength of binding of Mn^2+^ to each of the electrolyte components should not change, and the nature of the Mn^2+^ complexes and τ_M_ values should also remain unchanged. That is, in the pristine solutions, the only reason for a change in the relaxation rate is a change in *f*_M_, specifically arising from a different Mn^2+^ concentration. However, in the heated electrolyte solution (Figure 5b), new degradation species were generated, with potentially different binding affinities for Mn^2+^. The ^1^H EC *R*_1_ and *R*_2_ values predicted a Mn concentration of 2.58 ± 0.07 mM, while ICP-OES revealed an actual concentration of 4.67 ± 0.05 mM (predictions based on the relaxation rates of PF_6_^–^ and EMC were also inaccurate, with the least accurate estimates arising from the ^19^F PF_6_^–^ relaxation). Assuming, for simplicity, a constant chemical exchange correlation time in the pristine and degraded electrolyte solutions, the measured ^1^H EC *R*_1_ should only change when a change in *f*_M_ (the *x* variable in eqs 7 and 8) occurs. In the case of the degraded electrolyte, based on the measured ^1^H EC relaxation rates, *f*_M,EC_ was small—not because there were fewer Mn^2+^ ions in solution (as the calibration would predict) but, we suggest, because there were fewer Mn^2+^ ions bound to EC. It is possible that the relaxation measurement predicts only 55% of the actual Mn concentration because the other 45% of Mn ions that are bound to EC in the pristine electrolyte solution are instead bound to another species in the degraded solution (such as PO_2_F_2_^–^). This is consistent with our previous work showing that the addition of different solvents to electrolyte samples containing dissolved transition metals may dramatically affect transverse relaxation enhancement of electrolyte components, presumably by altering the metal coordination shell.^128^ Preferential coordination of transition metals by PF_6_^–^ degradation species has also been previously proposed by us^128^ and by others;^72,140^ upcoming work will also explore transition metal coordination in detail using NMR and electron paramagnetic resonance (EPR) spectroscopy. Theoretically, if the solvation shell surrounding the 4.67 mM Mn (the concentration present in the degraded electrolyte solution) comprised 6 EC molecules, this would correspond to a total displacement of 12.6 mM EC, likely by 12.6 mM of one or several other species. This is not an unreasonable level of electrolyte degradation: to generate 12.6 mM of fluorophosphate degradation species would require the decomposition of only 1.3% of all LiPF_6_ in a solution of 1 M LiPF_6_ in 3:7 EC/EMC. That the relaxation rates in Figure 5 predict, on average, only 58% of the total Mn concentration measured by ICP-OES is therefore consistent with the proposal that electrolyte degradation species preferentially bind to Mn^2+^, reducing the fraction of EC, EMC, and PF_6_^–^ in the Mn^2+^ solvation shell. Notably, this assumes that all change in the relaxation rate derives from a change in the coordinated fraction of EC, and that the effect of exchange is negligible. This smaller coordinated fraction results in longer ^1^H EC, ^1^H EMC, and ^19^F PF_6_^–^ relaxation rates than those seen in pristine electrolytes with similar paramagnetic metal ion concentrations, lessening the applicability of the calibration dataset.

Although the metal quantification in degraded samples is subject to inaccuracies arising from small changes in the metal solvation shell, conversely, this indicates that NMR relaxometry may be applied toward understanding the solvation shell in samples with a constant metal concentration. Such an application is common in the biochemical determination of protein structures, where spin labels are attached to particular protein sites, and the resulting differential relaxation enhancement provides information about distances between spin labels and other nuclei.^141−143^ The use of NMR relaxometry to decipher metal coordination in battery electrolytes may offer new insights into dissolution and deposition processes; while it is beyond the scope of this work to analyze metal solvation in detail here, such an analysis will be presented in upcoming work.

That the quantification of transition metals is less accurate when degradation species affect the metal solvation shell is a complicating factor to using the NMR method because transition metal dissolution is inherently a degradation mechanism—in particular, one that may be enhanced by the acidification of the electrolyte solution^40,64,144^ and the release of singlet oxygen,^145^ which reacts with electrolyte components.^146^ The idea that transition metal dissolution occurs in batteries in a manner that leaves the electrolyte solution in pristine condition is not realistic in traditional LiPF_6_/carbonate solutions. Hence, metal quantification *via* relaxometry is likely most feasible in systems where metal dissolution is substantial and electrolyte degradation is minimal. Examples include cathode symmetric cells, cells with high-voltage anodes and/or dissolution-prone cathodes (especially if operated at moderate voltages, where dissolution may outpace electrolyte degradation), and cells with more thermally, chemically, and electrochemically stable electrolyte solutions.

The results in Figure 5c also show that diluting the heat-degraded sample with pristine electrolyte increases the applicability of the pristine calibration data, returning the electrolyte and the Mn^2+^ coordination shell to a state closer to that found in the original calibration electrolyte. Similarly, if both the pristine calibration solutions and the experimental samples are manipulated to produce the same transition metal coordination shell—such as by adding a compound that is known to chelate the dissolved metals, and the compound is probed by NMR in addition to the electrolyte components—then it may be possible to apply the calibration to the degraded experimental samples without issue. However, both of these proposed adaptations of the method alter the electrolyte sample, and diluting the samples with pristine electrolyte obviously increases the minimum metal concentration required in the original sample. These adaptations may therefore not be feasible for metal quantification in *operando* NMR cells. *Operando* quantification is also further complicated by the possibility of multiple dissolved oxidation states (*e.g.*, paramagnetic Mn^2+^ and Mn^3+^ or paramagnetic Co^2+^ and undetectable diamagnetic Co^3+^) or multiple dissolved metals (*e.g.*, paramagnetic Fe^2+^ or Fe^3+^ from the dissolution of stainless steel cell parts). Caution is therefore needed in adapting the *ex situ* results presented here for any *operando* work.

## Conclusions

In this work, ^7^Li, ^31^P, ^19^F, and ^1^H NMR relaxation rates were used to indirectly quantify the concentration of dissolved transition metals in lithium-ion battery electrolyte solutions. Calibrations with M(TFSI)_2_ salts showed that NMR relaxation rates are proportional to the metal concentration and may be used to determine the concentrations of dissolved paramagnetic metals, with the Mn(TFSI)_2_ salt inducing the same relaxation behavior as Mn^2+^ dissolved from LiMn_2_O_4_ at ∼20 °C. In our solutions, ^1^H and ^19^F relaxation rates are more sensitive than ^31^P and ^7^Li relaxation rates to metal concentration, and Mn^2+^ induces faster relaxation rates than Co^2+^, Ni^2+^, or Cu^2+^; hence, the method is most sensitive to Mn^2+^. However, it is important that the solution composition and metal oxidation state are known and constant between the calibration and experimental samples: changes in the transition metal solvation shell, which may occur due to electrolyte degradation, impede the accuracy of relaxometric quantification. Relaxation rates also contain information about transition metal solvation, which may be another promising application of the method. Understanding transition metal dissolution behavior, including factors that enhance dissolution, may permit optimization of strategies to mitigate capacity losses induced by transition metal dissolution–migration–deposition processes.

## Supporting Information Available

Expanded view of ^7^Li and ^31^P relaxation; relationship between determination coefficient and relaxivity in Figures 1 and 2; limit of detection results with scale extended to 0.5 mM Mn^2+^; tabulated relaxation rates of samples containing 0, 0.001, and 0.005 mM Mn^2+^; and discussion of second LiMn_2_O_4_ experiment (PDF)
