# Supplementary material for: Quantifying Dissolved Transition Metals in Battery Electrolyte Solutions with NMR Paramagnetic Relaxation Enhancement
Source: J Phys Chem C Nanomater Interfaces. 2023 May 16;127(20):9509–21. doi: 10.1021/acs.jpcc.3c01396 (PMC10226131; doi:10.1021/acs.jpcc.3c01396)
Supplement: Supplementary file 1 — jp3c01396_si_001.pdf [file jp3c01396_si_001.pdf]

Supporting Information

Quantifying Dissolved Transition Metals in Battery Electrolyte Solutions with NMR Paramagnetic Relaxation Enhancement

Jennifer P. Allen,[1,2] Christopher A. O'Keefe,[1,2] Clare P. Grey[1,2]

1. Yusuf Hamied Department of Chemistry, University of Cambridge, Lensfield Road, Cambridge, CB2 1EW, Cambridge, UK.
2. The Faraday Institution, Quad One, Harwell Science and Innovation Campus, Didcot OX11 0RA, UK.

Figure S1 shows an expanded view of the ^{7}Li and ^{31}P relaxation data presented in Figure 1.

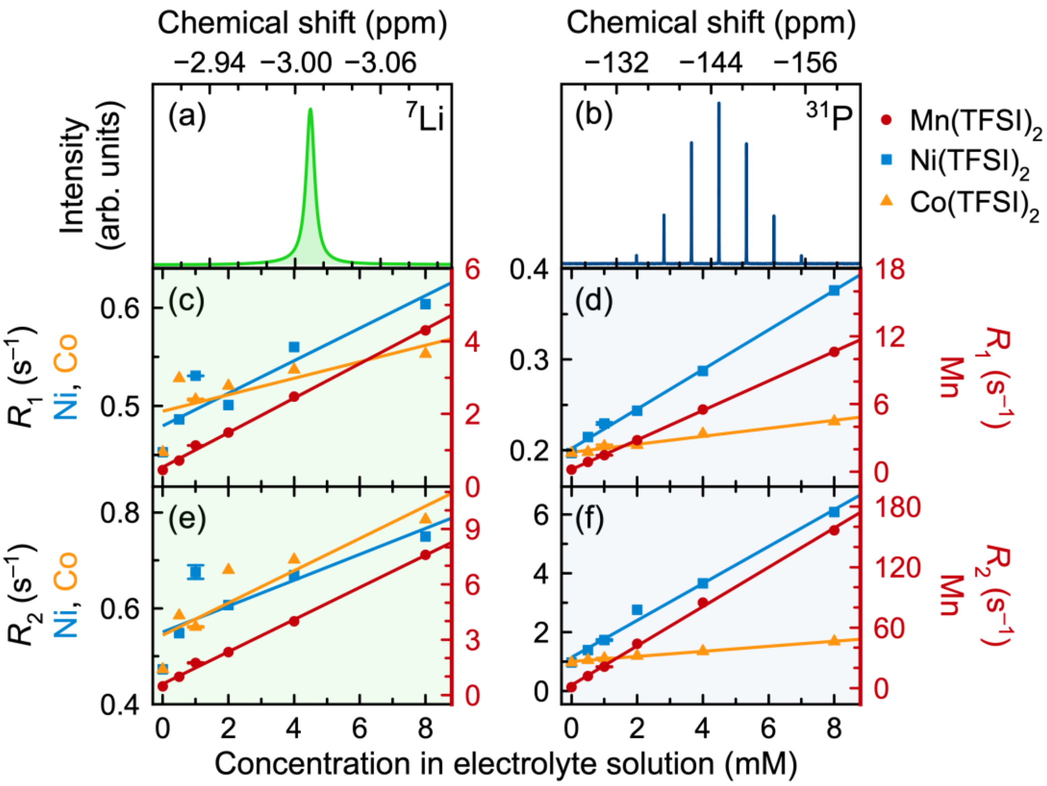

Figure S1. (a–b) ^{7}Li and ^{31}P representative NMR spectra of diamagnetic 1 M $LiPF_6$ in 3:7 EC:EMC (v/v), and (c–d) longitudinal and (e–f) transverse relaxation rates of electrolyte solutions containing dissolved $Mn(TFSI)_2$, $Ni(TFSI)_2$, or $Co(TFSI)_2$. Relaxation rates of solutions containing $Ni(TFSI)_2$ and $Co(TFSI)_2$ are plotted on the left *y* axes, while relaxation rates of solutions containing $Mn(TFSI)_2$ are plotted on the right *y* axes. Error bars at 1 mM indicate the standard deviation of three measurements on the sample.

Figure S2 shows the relationship between the gradient of the lines of best fit from Figures 1 and 2, and the determination coefficients of those lines of best fit. As the gradient increases

(*i.e.*, as the relaxation enhancement is larger) the quality of the fit is improved. Although not highlighted in this figure, it is also noted that linear fits of the R_1 concentration dependence are generally better than the R_2 fits, with determination coefficient values closer to 1. The T_2 measurement may be subject to additional error because the echo train that is used to refocus the magnetisation as part of the CPMG pulse sequence magnifies any imprecisions that may arise from using a non-180° pulse, whereas the inversion recovery method is less sensitive to a non-optimised pulse.

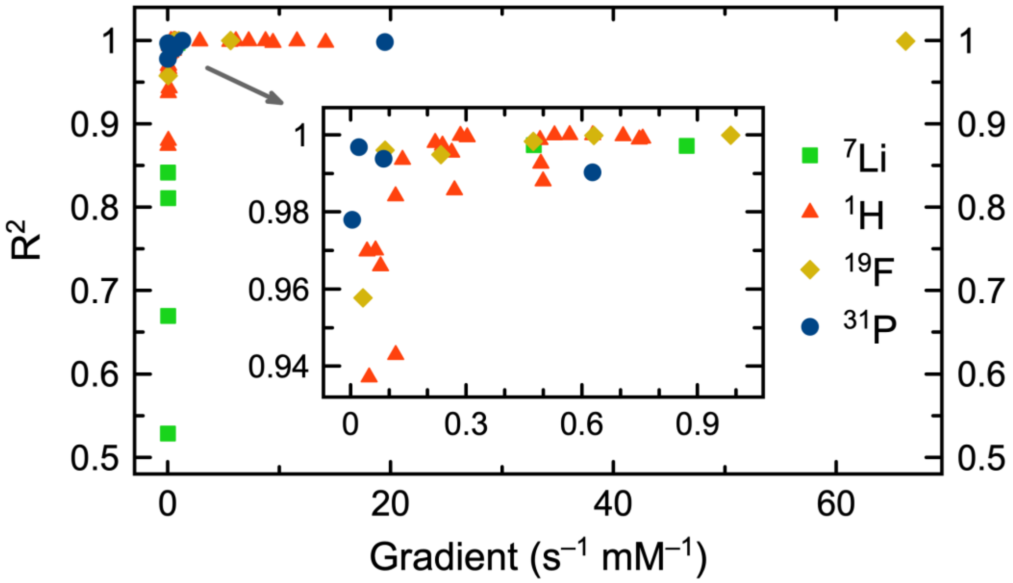

Figure S2. Gradients of all lines of best fit in Figures 1 and 2 plotted against the determination coefficients (R^2) of those lines of best fit. Gradients indicate the relationship between relaxation rates (R_1 or R_2) and transition metal concentrations.

Figure S3 shows an expanded view of the data presented in Figure 4, where relaxation rates are now shown for Mn^{2+} concentrations up to 0.5 mM. ^1H and ^{19}F relaxation rates at the 0, 0.001, and 0.005 mM Mn^{2+} points are given in Table S1.

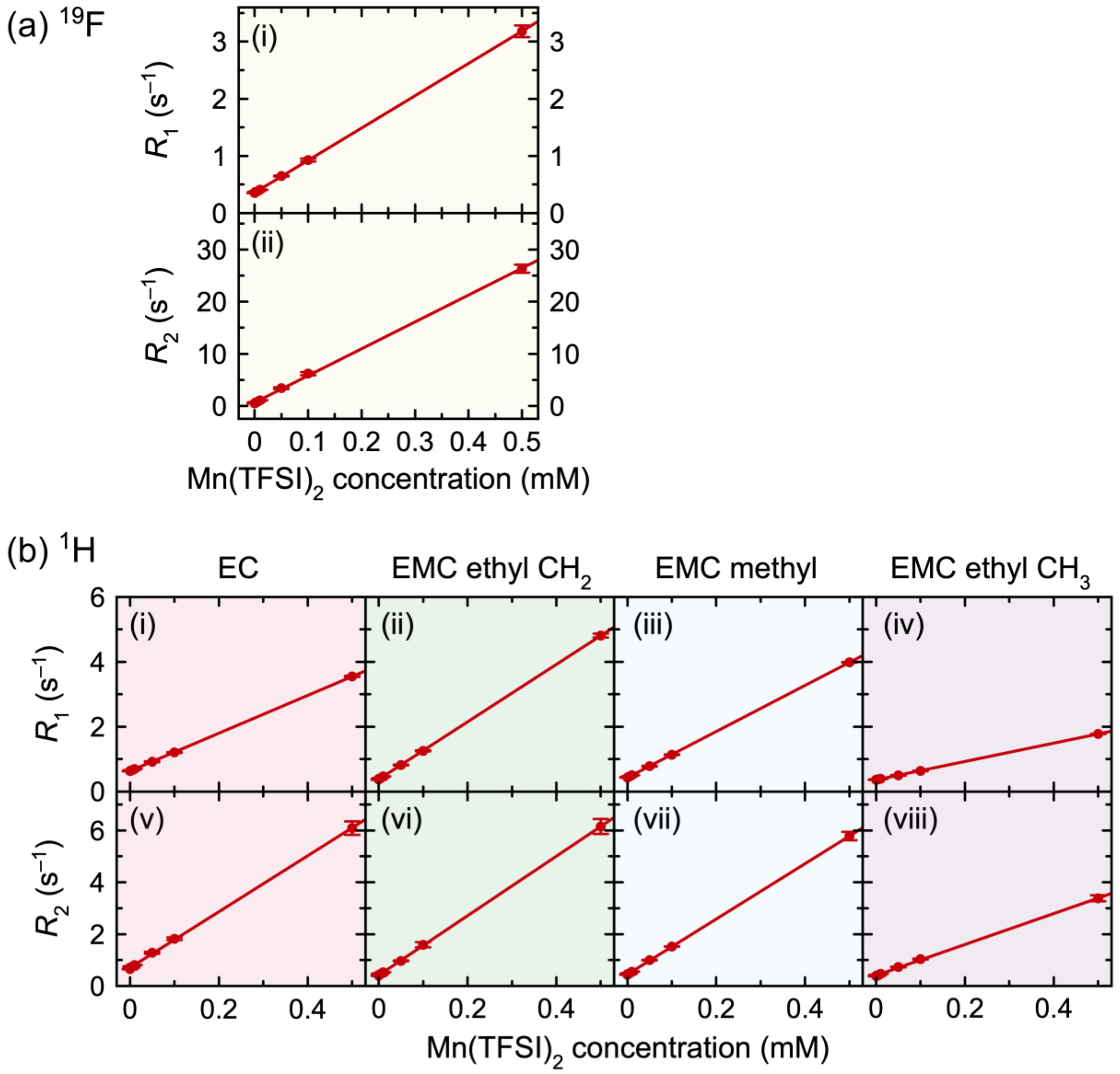

Figure S3. (a) ^{19}F and (b) ^{1}H longitudinal and transverse relaxation rates of 1 M $LiPF_6$ in 3:7 EC:EMC (v/v) containing small amounts of dissolved $Mn(TFSI)_2$ to determine the limit of detection of the method. The multiple relaxation rates in the ^{1}H NMR arise from the different ^{1}H environments in the solvent system.

Table S1. ^{19}F and ^{1}H relaxation rates of 1 M $LiPF_6$ in 3:7 EC:EMC (v/v) containing 0, 0.001, and 0.005 mM Mn^{2+}.

		0 mM Mn	0.001 mM Mn	0.005 mM Mn
^{19}F	R_1 (s^{-1})	0.352 ± 0.003	0.369 ± 0.004	0.380 ± 0.002
	R_2 (s^{-1})	0.4820 ± 0.0009	0.62 ± 0.08	0.77 ± 0.02
^{1}H EC	R_1 (s^{-1})	0.6307 ± 0.0002	0.64 ± 0.01	0.662 ± 0.002
	R_2 (s^{-1})	0.65 ± 0.02	0.72 ± 0.03	0.74 ± 0.03
^{1}H EMC ethyl CH_2	R_1 (s^{-1})	0.37 ± 0.01	0.40 ± 0.01	0.419 ± 0.008
	R_2 (s^{-1})	0.40 ± 0.01	0.44 ± 0.04	0.47 ± 0.02
^{1}H EMC methyl	R_1 (s^{-1})	0.431 ± 0.002	0.44 ± 0.01	0.462 ± 0.005
	R_2 (s^{-1})	0.4332 ± 0.0006	0.47 ± 0.02	0.49 ± 0.02
^{1}H EMC ethyl CH_3	R_1 (s^{-1})	0.3628 ± 0.0005	0.368 ± 0.006	0.376 ± 0.002
	R_2 (s^{-1})	0.39 ± 0.01	0.41 ± 0.02	0.42 ± 0.02

Figure S4 shows estimates of Mn concentration derived from the relaxation of electrolytes that were stored with $LiMn_2O_4$ (4 g $LiMn_2O_4$ + 3 mL electrolyte, stored 85 days in an argon glovebox).

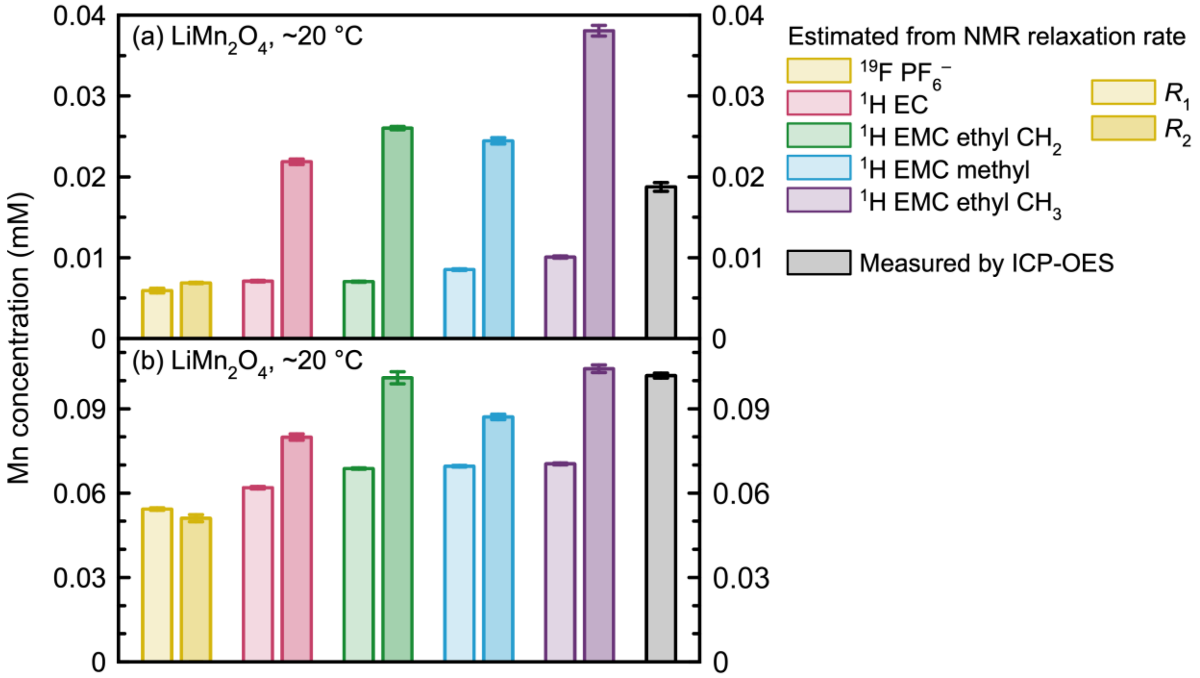

Figure S4. Estimated Mn concentrations after storage of $LiMn_2O_4$ with 1 M $LiPF_6$ in 3:7 EC:EMC (v/v), calculated from ^{19}F and ^{1}H longitudinal and transverse relaxation rates. Black bars indicate the concentration measured by ICP-OES, with error bars indicating the standard deviation of three samples. These measurements were performed 9 months after the calibration measurement was performed.

The estimates of Mn concentration in Figure S4 are less accurate than those obtained in Figure 5a; this difference is largely attributed to the nine-month gap that passed before measurement of the samples in Figure S4. It is anticipated that this difference arises due to (i) differences in the magnetic field, particularly as no field locking was used for relaxation measurements and/or (ii) differences in the electrolyte composition between the original calibration set and the experimental samples. For example, as a bottle of 1 M $LiPF_6$ in 3:7 EC:EMC is used, slow decomposition of PF_6^- to $PO_2F_2^-$ and HF occurs. The electrolyte used in these experiments may therefore not be 'pristine' in the same manner as the electrolyte used in the calibration set and the original experimental set. Also, in Figure S4a, it is also observed that the total Mn concentration is extremely small, which would be expected to introduce error into the result. If the relaxation method is to be used, these results suggest that regular calibration measurements should be performed to account for these changes. This is not unreasonable for an analytical method, but does increase the time required to measure a sample.
